# Distinct Patterns of P1 and C2 VEP Potentiation and Attenuation in Visual Snow: A Case Report

**DOI:** 10.3389/fneur.2021.723677

**Published:** 2021-11-17

**Authors:** Alison M. Harris

**Affiliations:** Department of Psychological Science, Claremont McKenna College, Claremont, CA, United States

**Keywords:** visual snow syndrome, visual evoked potentials, C2, habituation, double-pulse adaptation

## Abstract

Visual snow syndrome, characterized by persistent flickering dots throughout the visual field, has been hypothesized to arise from abnormal neuronal responsiveness in visual processing regions. Previous research has reported a lack of typical VEP habituation to repeated stimulus presentation in patients with visual snow. Yet these studies generally used pattern-reversal paradigms, which are suboptimal for measuring cortical responses to the onset of foveal stimulation. Instead, these responses are better indexed by the C2, a pattern-onset VEP peaking 100–120 ms after stimulus onset. In this case study, we analyzed the C2 and its adaptation profile in data previously collected from a single patient with visual snow using a “double-pulse” presentation paradigm. In controls, shorter intervals between stimulus pairs were associated with greater attenuation of the C2 VEP, with recovery from adaptation at longer stimulus onset asynchronies (SOAs). However, the visual snow patient showed the opposite pattern, with *reduced* C2 amplitude at longer SOAs despite distinct C2 peaks at the shortest SOAs. These results stand in contrast not only to the pattern of C2 VEP attenuation in controls, but also to a lack of adaptation previously reported for the pattern-onset P1 VEP in this patient. Exploratory source localization using equivalent current dipole fitting further suggested that P1 and C2 VEPs in the visual snow patient arose from distinct sources in extrastriate visual cortex. While preliminary, these results support differential patterns of VEP attenuation and potentiation within the same individual, potentially pointing toward multiple mechanisms of abnormal neuronal responsiveness in visual snow syndrome.

## Introduction

Visual snow syndrome is a debilitating neurological condition characterized by the persistent and continuous experience of tiny flickering dots in the visual field (1). Similar to migraine aura (2), the visual disturbances in visual snow syndrome have been hypothesized to arise from changes in neuronal responsiveness to sensory stimulation (3). Habituation, the phenomenon of reduced responsiveness over repeated stimulus presentation, is commonly observed in measurements of visual evoked potentials (VEPs) for healthy individuals (4). In contrast, loss of VEP habituation has been reported both in migraine with aura (5) and visual snow syndrome (6, 7).

Yet prior measurements of VEP habituation in visual snow have largely utilized pattern-reversal paradigms, in which a patterned stimulus switches polarity repeatedly over time. While pattern-reversal stimulation produces a reliable and consistent VEP across participants, the *pattern-reversal* P100^1^, this protocol is limited in terms of the cortical activity it represents. Specifically, the pattern-reversal P100 appears to largely reflect neural responses to the offset, rather than the onset, of the stimulus (8, 9), with contributions from both magnocellular and parvocellular pathways (10).

As a consequence, pattern-reversal paradigms may fail to capture cortical responses to the onset of foveal stimulation, information that is carried by parvocellular pathways of macular origin. Previous research suggests that these responses are indexed by the C2 (or CII) VEP elicited by pattern onset (11). Typically observed as a negative deflection peaking between 100 and 130 ms after stimulus onset (11–13), the C2 shows maximal amplitude for foveal stimuli with energy at high spatial frequencies (10). Source localization analyses suggest that this VEP arises adjacent to striate cortex (12), near the juncture of V1 and V2 (13).

Because the C2 is not visible in the pattern-reversal VEP (8), the effects of visual snow syndrome on this response have received little attention. However, in a recent study we recorded pattern-onset VEPs from a patient with visual snow (14). Our paradigm involved the central presentation of complex patterned stimuli with energy at high spatial frequencies, eliciting a strong C2 response. Additionally, we could measure the effect of repeated stimulus presentation through our use of a “double-pulse” presentation paradigm, in which two stimuli (S1 and S2) are presented with a varying stimulus onset asynchrony (SOA).

Double-pulse presentation has previously been linked to attenuation of pattern-onset VEPs (15–18), reflecting increased gamma oscillations in local inhibitory networks (19). This reduction is not explained by mere adaptation to low-level physical stimulus properties (18, 20), and is maximal at shorter SOAs (16, 17) when gamma power is highest (19). These properties distinguish double-pulse adaptation from pattern-reversal VEP habituation, which varies with stimulation parameters (e.g., reversal rate, check size, contrast) and is often strongest after successive blocks of stimulation (5, 21).

In our previous analysis, we replicated double-pulse adaptation of the *pattern-onset* P1 in normal individuals (14). In contrast, the patient with visual snow displayed a consistent pattern of P1 VEP potentiation, or enhancement, associated with decreased gamma-band inhibition, suggesting a physiological basis for VEP potentiation in visual snow (14). Although the neural mechanisms of pattern-onset P1 enhancement in this paradigm potentially differ from those underlying reduced VEP habituation in visual snow (6, 7), our data are nonetheless broadly consistent with increased excitability of visual cortex in this condition.

Here we performed a novel analysis quantifying the C2 response and its double-pulse adaptation profile in data previously collected from a patient with visual snow syndrome (14). Based on other research on attenuation of the C2 response, we predicted that in normal individuals there would be C2 adaptation to the S2 stimulus at SOAs <80 ms, with recovery from adaptation for SOAs of 100 ms and above (16). We could then test whether the patient with visual snow showed a similar pattern of attenuation to controls. Finally, due to our use of a high-density EEG array, we were able to separate signals corresponding to the C2 from the previously-reported P1 response. This enabled us to directly compare the pattern-onset C2 and P1 components, providing further insight into how the cortical responses indexed by these two VEPs may vary.

## Method

### Participants

Because this is a re-analysis of an existing dataset, participants and methods are the same as previously described in the 2018 study by Luna, Lai, and Harris (14). At the time of testing, the patient was a right-handed male (age 22 years) with a 2-year history of visual snow syndrome. In line with diagnostic criteria for visual snow (1), he reported experiencing constantly flickering fine dots throughout his visual field which persisted across light conditions without remission, along with palinopsia, nyctalopia, photopsia, phosphenes, the blue field entopic phenomenon, and tinnitus. The patient had a family history of migraine with aura on the maternal side, and reported one previous episode of migrainous phenotype with symptoms of migraine aura 6 years prior to the time of testing. However, no other migraine attacks were reported, discounting episodic migraine as a factor in the patient's symptoms. Measurements of visual acuity and eye structure were normal, and neurological and neuroimaging examinations found no abnormalities.

Seven control participants with normal or corrected vision were recruited from the college community. Controls were matched to the patient on gender and age (ages 20–24, mean age = 21.1), but reported no personal or family history of migraine. Three of these participants were excluded due to problems with EEG recording (*n* = 2) and failure to identify sensors displaying a C2 response within the predefined time window of interest (*n* = 1). Thus, 4 control participants were included in the final analysis, a sample size in line with prior studies of the C2 component (11). Informed consent was obtained from all participants, and the study was approved by the Claremont McKenna College Institutional Review Board.

### Materials and Methods

**Figure 1:**
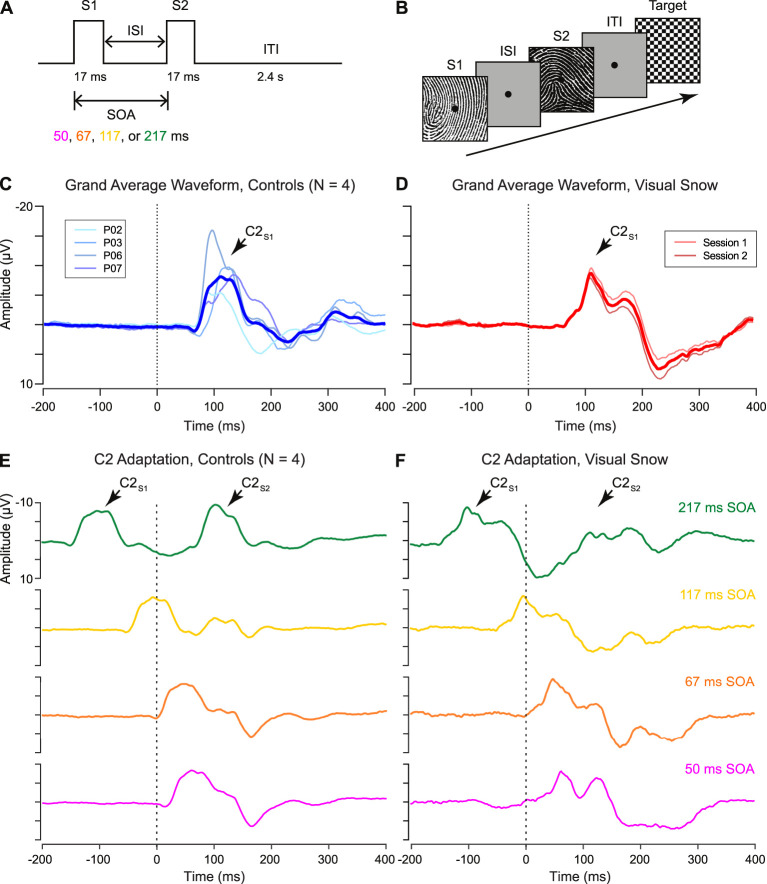
**(A)** Schematic of “double-pulse” presentation paradigm. Two stimuli (S1 and S2) are presented with a variable interstimulus interval (ISI), resulting in a stimulus onset asynchrony (SOA) of 50, 67, 117, or 217 ms. After the S2 stimulus, an intertrial interval (ITI) of 2.4 s was selected to minimize persistent afterimages in the visual snow patient. **(B)** Sample trial structure. On each trial, a high-contrast stimulus was selected to serve as S2, paired with a randomly selected non-identical image as S1. Participants monitored for the appearance of an infrequent target (checkerboard pattern), which occurred on 10% of trials. **(C,D)** Grand average waveform for C2 response in **(C)** controls and **(D)** visual snow patient, as identified from the unadapted S1 condition. **(E,F)** Adaptation of the C2 response in **(E)** controls and **(F)** visual snow patient for each tested SOA (green: 217 ms, gold: 117 ms, orange: 67 ms, fuschia: 50 ms). Grand average waveforms are time-locked to the onset of the S2 stimulus (dotted line, 0 ms), with the C2 response visible ~100 ms after stimulus onset. Grand averages in **(F)** reflect the average of two separate sessions in the visual snow patient.

Figure 1A shows the double-pulse stimulus presentation paradigm from the 2018 study by Luna, Lai, and Harris (14). On each trial, two stimuli were presented in brief succession with a variable intertrial interval ranging from 33 to 200 ms. Each stimulus was displayed for 17 ms, resulting in a stimulus onset asynchrony (SOA) of 50, 67, 117, or 217 ms. Stimuli consisted of 50 high-contrast black-and-white line (fingerprint) patterns (4.6° × 4.6° of visual angle) displayed on a gray background with a central fixation point (Figure 1B). Each pattern served as the S2 stimulus twice per condition (100 trials per condition), with a non-identical image randomly selected on each trial to serve as S1. Participants were instructed to respond by keypress to the appearance of an infrequent target stimulus, a checkerboard pattern, which occurred in 10% of the total trials. Target trials were randomly intermixed with experimental double-pulse trials, and all double-pulse presentation conditions were randomly interleaved within participants. The experiment was programmed and displayed in Matlab (Mathworks, Natick, MA) using PsychToolbox (22) stimulus presentation software.

Control participants each completed a single session of testing with all four SOA conditions, for a total of 400 trials per participant. To verify that the VEP response observed in the patient reflected a consistent pattern, he participated in two separate recording sessions ~1 month apart. All statistical analyses were performed for data averaged across both sessions.

### EEG Data Acquisition and Preprocessing

Continuous EEG data were collected using a 128-channel BioSemi ActiveTwo system (Biosemi B.V., Amsterdam, Netherlands). Data were digitized at 512 Hz with bilateral mastoid references. Offline data processing was performed in the EEGLAB toolbox (23). Data preprocessing steps included resampling to 500 Hz, re-referencing to an average reference, linear detrending, high-pass filtering at 1 Hz, notch filtering at 60 Hz, extraction of epochs time-locked to S1 (−500 to 800 ms), and removal of artifactual noise via independent components analysis (24) using second-order blind identification (25, 26). Finally, 600-ms epochs time-locked to the S2 stimulus (−200 ms to 400 ms) were extracted for analysis.

### VEP Data Analysis

VEP waveforms were extracted from the EEG recording by averaging time-locked signals across trials in each condition. The C2 component of the VEP was defined as a negative deflection occurring ~100–120 ms after stimulus onset at posterior sensors (Figures 1C,D). Sensors of interest (SOIs) were defined individually for each participant 100–120 ms post-stimulus onset at posterior sensors based on the amplitude of VEPs to the S1 stimulus, using a threshold of z-scored amplitude ≤-1.5. Local peak amplitude and latency for the S2 response were then determined for each participant and condition using a 10-point (20-ms) window in the ERPLAB (27) toolbox for Matlab. Amplitude of the C2 response to the S2 stimulus was normalized by the amplitude of the preceding S1 response (C2_S2_/C2_S1_) to quantify attenuation and/or potentiation of the second C2 response. To examine the trial-by-trial variability in the C2 VEP, we identified the independent component (IC) associated with the negative C2 deflection at midline occipital sensors from one session in the visual snow patient and a representative control participant.

In order to verify that the pattern of double-pulse adaptation for the C2 VEP was distinct from that for the previously-described P1 component, it was necessary to directly compare the current results to normalized amplitude values derived from our prior study (14). Here we focused only on the two extreme conditions (50 vs. 217 ms SOA), further identifying a separate IC that showed a scalp topography and average waveform consistent with the pattern-onset P1 component. Although by necessity these results build on data previously reported in a separate publication (14), these secondary analyses are largely based on a different analytical approach, with the goal of providing complementary information to our original analysis.

Finally, to shed light on the neural sources of the C2 response, dipole fitting was applied using the DIPFIT plugin in EEGLAB. Equivalent current dipoles were fit to ICs associated with the pattern-onset C2 and P1 scalp VEPs from one session in the visual snow patient. A template boundary element model (BEM) based on the MNI brain was used for the head model, with manual co-registration of the EEG electrode locations to the head model. The appropriate ICs for dipole fitting were identified based on scalp topography and average waveform responses, and then fit via a two-step iterative process in EEGLAB, consisting of an initial coarse grid search followed by a fine-grain fitting via a non-linear optimization algorithm. The number of dipoles and symmetry constraint for each IC were determined based on minimizing residual variance (RV), while the dipole moment ratio (DMR) was checked to ensure that both dipoles contributed to fitted models with two dipoles (28).

## Results

Although the polarity of the C2 varies depending on which hemifield is visually stimulated, it has typically been reported as a negative deflection emerging from 100 to 130 ms after stimulus onset (11), perhaps due to superposition with the N1 component in the same time window (13). Examining the response to the S1 stimulus, we successfully identified a VEP matching these parameters in 4 control participants (Figure 1C), as well as in the patient with visual snow (Figure 1D). As shown by the individual waveforms plotted in Figure 1C, the C2 component recorded at the scalp showed substantial individual variation in terms of its amplitude and latency. Nonetheless, no differences in amplitude were observed between the C2_S1_ component in controls and the visual snow patient (Table 1), as evidenced by a one-sample *t*-test [*t*_(3)_ = −0.54, *p* = 0.63]. Likewise, latency of the C2_S1_ response was similar across controls and the visual snow patient (Table 1), and not significantly different between the groups [*t*_(3)_ = −0.31, *p* = 0.78].

**Table 1:** C2 component in controls vs. visual snow.

	**Controls (*N* = 4)**	**Visual snow (*n* = 2)**	***P*-value**
Average amplitude (μV) ± SD	−9.89 ± 4.44	−8.7 ± 1.2	0.63
Amplitude range (μV)	−5.43 to −16.0	−7.85 to −9.55	
Average peak latency (ms) ± SD	109.7 ± 21.6	113 ± 1.41	0.78
Latency peak range (ms)	88–132.3	112–114	

*Average amplitude and latency of the C2 component in 4 controls compared to the visual snow patient (2 sessions). Although data from both sessions in the visual snow patient were averaged for statistical analysis, the standard deviation and range are presented here to demonstrate the high consistency of the C2 component in the patient across experimental sessions*.

Next, we quantified the C2 response to double-pulse stimulus presentation across varying SOAs in controls (Figure 1E). In line with previous findings, the controls showed a pattern of decreasing adaptation at longer SOAs (16), with the C2_S2_ responses at shorter SOAs of 50 and 67 ms appearing partially integrated with the initial C2_S1_ response. At the shortest SOA, positive average C2_S2_ amplitude (5.99 ± 4.31 μV) reflected a 160.5% decrease relative to C2_S1_. However, at a SOA of 117 ms, average C2_S2_ amplitude was still attenuated (−3.02 ± 4.61 μV, 69.5% decrease), in contrast to our earlier findings for the P1_S2_ response (14). Recovery was only complete by the longest SOA (−10.9 ± 4.69 μV). Therefore, even within the neurotypical brain, the pattern-onset P1 and C2 components may be distinguished not only by their retinotopic organization (13), but also by their double-pulse adaptation profiles.

A very different pattern was observed for the patient with visual snow (Figure 1F). At the shortest SOAs, associated with complete or partial integration of the C2_S2_ response in controls, the patient showed a clear double peak, suggesting a relative lack of attenuation (50 ms SOA: mean = −6.4 ± 1.58 μV, 26.4% decrease). Conversely, for the longest SOA of 217 ms—associated with complete recovery in controls—the patient's C2_S2_ peak was dramatically reduced (mean = −2.93 ± 0.97 μV, 66.3% decrease). Thus, longer intervals between stimulus presentations produce abnormal adaptation of the C2 response in this patient. One clue to the origin of this effect comes from the strong positive deflection following the patient's C2 component 200–300 ms post-stimulus onset (Figure 1D). Strikingly, this peak for the *unadapted* C2_S1_ response in the patient parallels the pattern of attenuation at short SOAs in controls (Figure 1E), who show a positive deflection 150–200 ms post-stimulus onset in lieu of the negative C2_S2_ response.

**Figure 2:**
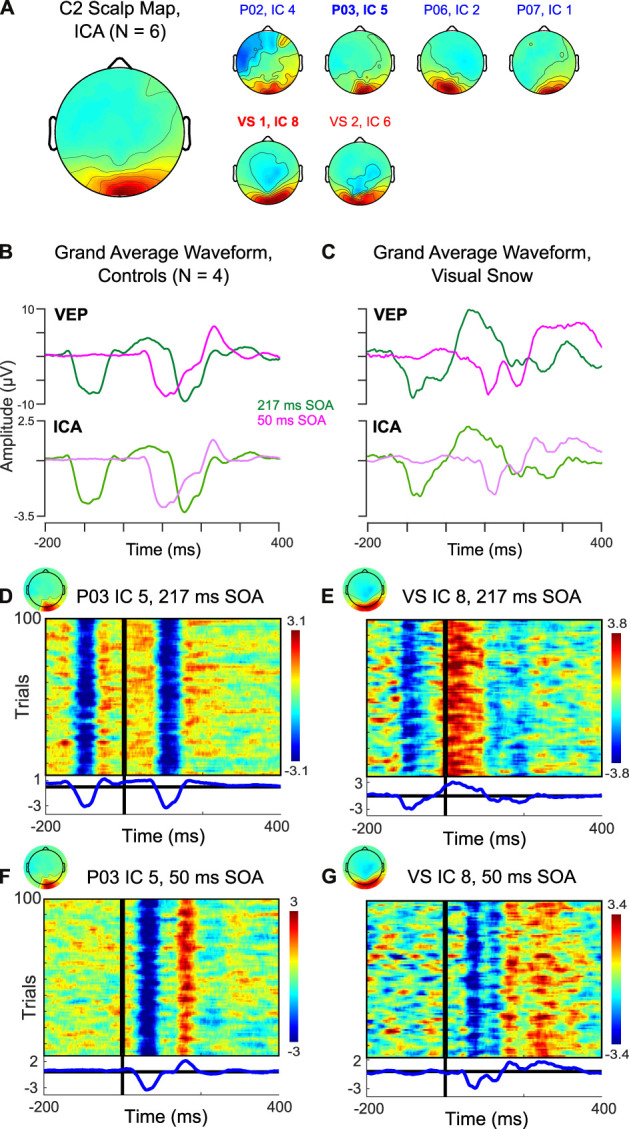
Independent component analysis (ICA) of C2 response. **(A)** Scalp topography associated with C2 IC across 4 controls (blue) and 2 sessions in the visual snow patient (red). **(B,C)** Comparison of back-projected grand average waveform from ICA to C2 VEP measured at the scalp in **(B)** controls and **(C)** visual snow patient. **(D–G)** Plots of trial-by-trial activity associated with C2 IC in **(D)** a representative control participant at 217 ms SOA, **(E)** the visual snow patient at 217 ms SOA, **(F)** a representative control participant at 50 ms SOA, and **(G)** the visual snow patient at 50 ms SOA.

However, one potential confound arises from the fact that the scalp VEP may reflect the superposition of multiple cortical sources, particularly in the short time frame of early visual processing. To address this issue, previous research has used independent component analysis (ICA) to separate the C2 component from other early VEPs (13). For each participant, we identified an independent component (IC) corresponding to the C2 response, which was distributed over midline occipital electrodes (Figure 2A). In controls, the average waveform obtained from back-projecting these ICs showed a clear negative deflection ~100–120 ms after stimulus onset in the 217 ms SOA condition (Figure 2B, top), in line with the VEP observed at the sensor level (Figure 2B, bottom). Thus, the selected ICs appear to satisfactorily capture the C2 VEP measured at the scalp. Trial-by-trial data from a single representative participant indicate that the average waveforms reflect highly consistent peaks in the data which are reliably time-locked to the stimulus (Figure 2D), including the positive deflection associated with rapid double-pulse presentation in the 50 ms SOA condition (Figure 2F).

Figure 2C compares the IC back-projected average and scalp VEP for our single patient with visual snow. Notably, the IC data preserves the pattern of a large positive deflection following the S1 stimulus in the 217 ms SOA condition. Likewise, the double peak in the 50 ms SOA condition is present, albeit somewhat reduced. These results further support the idea that differences in the C2 response observed in this particular visual snow patient arise from changes in the response properties of this VEP's neural generators, rather than from a superposition of multiple sources in the visual association cortex. Visualization of the trial-by-trial IC data for the 217 ms (Figure 2E) and 50 ms SOA conditions (Figure 2G) in one session underscores that these waveforms are highly consistent from trial to trial, making it unlikely that the distinctive characteristics of the C2 in this individual reflect a small number of outlying trials.

**Figure 3:**
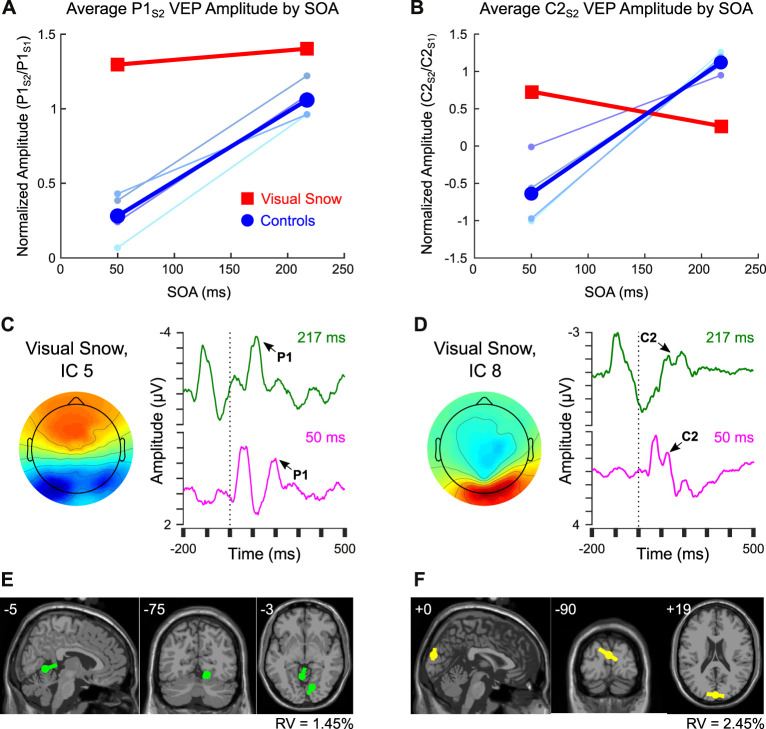
Comparison of pattern-onset P1 and C2 adaptation effects. **(A,B)** Sensor-level VEP amplitude for 50 ms and 217 ms SOA in controls (blue) compared to the visual snow patient (red) for **(A)** the P1 response vs. **(B)** the C2 response. Colors for individual control participants correspond to labels from Figure 1C. **(C,D)** Comparison of ICs from one session within the visual snow patient associated with the **(C)** P1 vs. **(D)** C2 VEPs, including scalp topography and back-projected grand average waveform for 217 ms SOA (green) and 50 ms SOA (fuschia). **(E,F)** Equivalent current dipole fitting for **(E)** IC 5 associated with the P1, and **(F)** IC 8, associated with the C2 response.

These data provide preliminary evidence for distinct patterns of attenuation for the C2 VEP in normal controls compared to a patient with visual snow syndrome. The pattern of C2 VEP adaptation in this patient with visual snow also appears to differ dramatically from the potentiation observed across SOAs for the pattern-onset P1 response in a previous analysis (14). Whereas the visual snow patient showed sustained potentiation of the P1 VEP across the shortest and longest SOAs, the same individual evinced a reduction of C2 amplitude at the longer SOA (Figures 3A,B, red squares). In contrast, controls consistently showed increases in amplitude with increasing SOAs (Figures 3A,B, blue circles), in line with previous reports (15, 16). To quantify these effects, we computed the difference in amplitude of the P1 and C2 components at 217 vs. 50 ms SOAs, then calculated the difference of differences to determine how the two components varied from each other [(P1_Long_-P1_Short_)—(C2_Long_-C2_Short_)]. Comparing the values of the control group to the visual snow patient using a one-sample *t*-test revealed a significant effect [*t*_(3)_ = −4.59, *p* = 0.019], reflecting a disparity between controls (mean P1-C2 difference score = −0.98 ± 0.67) and the visual snow patient (P1-C2 difference score = 0.57), largely driven by the patient's reduced C2 attenuation at short SOAs. Thus, the adaptation profiles for the pattern-onset P1 and C2 VEPs observed at the sensor level appear to be distinct within a single patient with visual snow syndrome.

Finally, previous research suggests that the C2 may be part of a VEP complex distinct from the pattern-onset P1 response, both in terms of retinotopic organization and putative neural sources (13). To test this idea in our data, we identified ICs associated with the pattern-onset P1 (IC 5, Figure 3C) vs. C2 (IC 8, Figure 3D) VEPs. As in the IC-level analysis above, the differential pattern of habituation between these two responses remained visible in VEPs back-projected from the selected ICs, suggesting that these results do not merely reflect superposition of multiple sources. An exploratory equivalent current dipole fitting analysis for both ICs (Figures 3E,F) found that two-dipole solutions produced the lowest residual variance (IC 5: 1.45%, IC 8: 2.45%). Dipole moment ratios near 1 (IC 5: DMR = 1.4, IC 8: DMR = 1.0) indicated that the decreased residual variance for each of these fits reflected meaningful contributions from both dipoles rather than overfitting of model noise. Critically, the dipole solutions for the two ICs indicated different neural sources. Whereas the IC associated with the P1 was localized to sources in the right ventral extrastriate cortex (MNI coordinates: 14, −75, −12) and left cerebellum (−5, −55, −2), the IC associated with the C2 VEP was best fit by symmetric dipoles originating from dorsal extrastriate cortex (0, −90, 19) oriented in opposite directions. Although these results should be interpreted with caution given the low spatial resolution of EEG, they provide preliminary support for the idea that C2 attenuation may reflect distinct neural mechanisms from the previously observed potentiation of the P1 VEP in this individual with visual snow.

## Discussion

Lack of VEP habituation in visual snow syndrome has been cited as evidence for the idea that this condition reflects altered neuronal responsiveness to sensory stimulation (6, 7). Yet the majority of these studies have relied on pattern-reversal presentation paradigms, which elicit strong pattern-offset VEPs thought to be driven by both magnocellular and parvocellular systems (8–10). Therefore, the role of cortical responses to foveal stimulus onset, as indexed by the C2 component (8, 10, 11), has been relatively unexplored in visual snow. In this study, we reanalyzed existing data using a double-pulse adaptation paradigm in order to quantify adaptation of the C2 response in neurotypical controls in comparison to a patient with visual snow syndrome.

The C2 was visible both in control participants and in the visual snow patient as a negative-going deflection ~100–120 ms after stimulus onset at midline occipital electrodes. As far as we know, this is the first reported characterization of this component in a patient with visual snow, suggesting that early pattern-onset VEPs can be identified in this neurological condition. However, the response properties of the C2 component differed dramatically between controls and the visual snow patient. Whereas controls showed increasing attenuation at shorter SOAs, in line with previous data (16), the visual snow patient displayed the opposite pattern, with a clear double peak at the shortest SOA but reduced amplitude at the longest SOA. This differential response was associated with an enhanced positive deflection following the C2_S1_ response, which was strongest at the 50 ms SOA in controls but most visible for the 217 ms SOA in the visual snow patient. Although the generalizability of results from a single patient is extremely limited, these results nevertheless provide a provisional first description of abnormal VEP responsiveness of the C2 component in association with visual snow symptoms. Given the putative role of parvocellular input in the C2 response (10), these results complement colorimetry findings attributed to imbalances in the koniocellular and/or magnocellular pathways in visual snow (3), possibly suggesting more general abnormalities across systems receiving input from the different visual pathways.

The adaptation profile of the C2 response in the visual snow patient clearly contrasted with the potentiation of his pattern-onset P1 response, described in a previous study (14). Exploratory dipole fitting in data from one recording session in the patient further suggested that the pattern-onset P1 and C2 VEPs in this individual may be localized to separate sources in extrastriate cortex. Specifically, the P1 response was associated with asymmetric dipoles in regions of the right ventral extrastriate cortex and left cerebellum. Interestingly, these coordinates were similar to those reported previously in a neuroimaging study of hypermetabolism in visual snow (29), though caution in comparing these methodologies is warranted given the poor spatial resolution of EEG. In contrast, the IC associated with the C2 VEP in this patient was best fit by symmetric dipoles originating from dorsal extrastriate cortex. Although equivalent current dipole fits rely on numerous assumptions, and should thus be interpreted with care, these results nonetheless join other studies supporting the idea that the C2 and P1 VEPs recorded at the scalp originate from distinct cortical sources (13).

Together, these results corroborate the idea that VEPs measured at the scalp may reflect heterogenous sources in the early visual processing stream (13), leading to the striking observation of differential attenuation or potentiation of the VEP response *within a single individual* with visual snow. Previous work suggests that the scalp VEP captures cortical responses both to increases and decreases in contrast, with positive “contrast decrease” signals at pattern offset contributing particularly to the pattern-reversal VEP (8). This “contrast decrease” response may be anomalous in patients with visual snow, contributing to previously-reported differences in pattern-reversal P100 amplitude (30) or habituation (6, 7) which may occur as part of, or in addition to, decreases in cortical inhibition following visual stimulation (14). At the same time, through its interaction with foveal “contrast increase” signals, this atypical “contrast decrease” component could explain the C2 attenuation observed here for a single visual snow patient. As the interval between stimuli decreases, the pattern-offset signal may be attenuated, resulting in the recovery of the C2 component at short SOAs. Further experiments directly comparing VEP components to pattern onset, offset, and reversal within patients with visual snow will be necessary to test this hypothesis.

One major caveat of the present study is that these data come from a single patient, limiting the generalizability of these results. Therefore, it is essential that these findings be replicated with a larger sample of patients, as well as including greater heterogeneity in terms of gender, age, and co-morbid factors such as migraine with aura. However, despite the exploratory nature of these results, they support using diverse experimental paradigms and stimulation protocols to assess cortical visual function in visual snow. While pattern-reversal VEP habituation is valuable given its extensive characterization at the clinical level, it depends on stimulation parameters (5), may be conflated with changes in attention and arousal (21), and shows high intra-individual variability (31). These factors may contribute to failures to replicate decreased habituation in migraine and visual snow [e.g., (21, 30, 32)]. Our data join other recent results (13) suggesting that VEPs may emerge from multiple cortical sources and reflect differential neural responses to pattern onset and offset. A more refined understanding of how these signals are perturbed in visual snow syndrome could help to shed light on how neuronal responsiveness of the visual processing stream is affected by this debilitating condition.

## Data Availability Statement

The raw data supporting the conclusions of this article will be made available by the authors, without undue reservation. Requests to access these datasets should be directed to Alison Harris, aharris@cmc.edu.

## Ethics Statement

The studies involving human participants were reviewed and approved by Claremont McKenna College Institutional Review Board. The patients/participants provided their written informed consent to participate in this study.

## Author Contributions

AH was responsible for conceiving of the analyses, analyzing and interpreting the data, and writing the manuscript.

## Conflict of Interest

The author declares that the research was conducted in the absence of any commercial or financial relationships that could be construed as a potential conflict of interest.

## Publisher's Note

All claims expressed in this article are solely those of the authors and do not necessarily represent those of their affiliated organizations, or those of the publisher, the editors and the reviewers. Any product that may be evaluated in this article, or claim that may be made by its manufacturer, is not guaranteed or endorsed by the publisher.

## Footnotes

^1^Although the terms “P100” and “P1” have both been applied to positive deflections 100 ms after stimulus onset in pattern-reversal and pattern-onset stimulation, here we refer to the former as the *pattern-reversal* P100 and the latter as the *pattern-onset* P1 so our findings can be more easily related to the existing literature.
